# Patients with shoulder syndromes in general and physiotherapy practice: an observational study

**DOI:** 10.1186/1471-2474-14-128

**Published:** 2013-04-08

**Authors:** Margit Kooijman, Ilse Swinkels, Christel van Dijk, Dinny de Bakker, Cindy Veenhof

**Affiliations:** 1 Department of Allied Health Care, NIVEL, Netherlands Institute of Health Services Research, PO Box 1568, Utrecht, BN, 3500, the Netherlands; 2 Scientific Centre for Transformation in Care and Welfare (TRANZO), Tilburg University, PO Box 90153, Tilburg, LE, 5000, the Netherlands

**Keywords:** Shoulder impingement syndrome, Physical therapy specialty, Primary health care

## Abstract

**Background:**

Shoulder complaints are commonly seen in general practice and physiotherapy practice. The only complaints for which general practitioners (GPs) refer more patients to the physiotherapist are back and neck pain. However, a substantial group have persistent symptoms. The first goal of this study is to document current health care use and the treatment process for patients with shoulder syndromes in both general practice and physiotherapy practice. The second goal is to detect whether there are differences between patients with shoulder syndromes who are treated by their GP, those who are treated by both GP and physiotherapist and those who access physiotherapy directly.

**Methods:**

Observational study using data from the Netherlands Information Network of General Practice and the National Information Service for Allied Health Care. These registration networks collect healthcare-related information on patient contacts including diagnoses, prescriptions, referrals, treatment and evaluation on an ongoing basis.

**Results:**

Many patients develop symptoms gradually and 35% of patients with shoulder syndromes waited more than three months before visiting a physiotherapist. In 64% of all patients, treatment goals are fully reached at the end of physiotherapy treatment. In general practice, around one third of the patients return after the referral for physiotherapy. Patients with shoulder syndromes who are referred for physiotherapy have more consultations with their GP and are prescribed less medication than patients without a referral. Often, this referral is made at the first consultation. In physiotherapy practice, referred patients differ from self-referrals. Self-referrals are younger, they more often have recurrent complaints and their complaints are more often related to sports and leisure activities.

**Conclusions:**

There is a fairly large group of patients with persistent symptoms. Early referral by a GP is not advised under current guidelines. However, in many patients, symptoms develop gradually and a wait-and-see policy means more valuable time may pass before physiotherapy intervention takes place. Meanwhile a long duration of complaints is a predictor for poor outcome. Therefore, future research into early referral is required. As physiotherapists, we should develop a way of educating patients to avoid lengthy waiting periods before seeking help. To prevent high costs, physiotherapists could consider a classification of pain and limitations and wait-and-see policy as used by GPs. With early detection, a once-off consultation might be sufficient.

## Background

Shoulder complaints are the most common complaints of the extremities in an average physiotherapy practice; 9.8% of all patients present with this type of problem [1]. Low back pain and neck pain are the only complaints for which a general practitioner (GP) refers more patients to the physiotherapist; 7.3% of all referrals to a physiotherapist are made for shoulder complaints [2]. Studies report unfavourable outcomes in many patients [3-5][6], high costs in terms of secondary care and sick leave [7] and frequent occurrence in the workplace [8]. Therefore, shoulder conditions involve a considerable burden for the individual and the society.

Shoulder complaints can be roughly divided into problems with the glenohumeral joint (frozen shoulder, osteoarthritis), shoulder instability, acromioclavicular or sternoclavicular complaints, cervical or cervicothoracic dysfunction and problems with structures in the subacromial space. It has been estimated that approximately 44-80% of all shoulder complaints originate from these anomalies of structures in the subacromial space [9][10]. This space contains the tendons of the rotator cuff muscles and two bursae. Entrapment or inflammation of these structures leads to a restricted range of motion and pain. Although these complaints are described as shoulder syndromes, there is lack of consensus on the diagnostic criteria and on the best approach to management [11][12][13][14]. Incidence and prevalence of shoulder conditions have been identified but there are no such estimates for shoulder syndromes [14]. In addition, a recent systematic review of the literature indicates that many studies on the management of impingement syndrome are deficient in detailed demographic information, as well as information on previous medical treatment such as corticosteroid injections or (non-) steroidal anti-inflammatory drugs, previous physiotherapy and even the duration of the symptoms at the start of treatment [14]. The current study provides this information for a large group of patients who consulted their general practitioner (GP) or physiotherapist for these complaints.

As referred to above, there is debate on the best treatment methods for patients with shoulder syndromes. Dorrestijn et al. [11] and Kromer et al. [15] showed that so far, there is no evidence indicating whether surgical treatment or conservative treatment has a better outcome for patients with shoulder syndromes. Therefore, it is suggested that patients should be treated conservatively before surgical intervention is considered [15]. There is a Dutch guideline for (general) shoulder complaints for GPs that suggests a stepwise approach of advice, analgesia and referral for physiotherapy [9]. There also is a short guideline available for physiotherapists. This is based partly on scientific evidence and partly on best practice because the content of physiotherapy treatment, as part of conservative treatment, is still under discussion [16]. This results in a variable number of patients (20 -79%) that respond well to physiotherapy [10]. In order to improve treatment, knowledge of current treatment methods is indispensable but as yet, it is not adequately available.

Using data from registration networks, the present study describes patient characteristics and the treatment process for patients treated by a representative group of GPs or physiotherapists who were unaware of the specific purpose of this study. In the Netherlands, patients can access physiotherapy professionals directly (known as direct access or self-referral) and it is known that use, treatment and outcome may differ depending on the mode of access [17]. However, it is not known whether this is also true for patients with shoulder syndromes specifically. By separating patients who were referred for physiotherapy from those who were not, an attempt was made to describe the care of the two groups and to determine if they were materially different. In brief, the present study addresses two research questions. First, what are the characteristics of the patient population and the care process in patients with shoulder syndromes in general and in physiotherapy practice in particular? Second, does the population and care process differ between patients treated solely by their GP, those referred for physiotherapy and self-referrals?

## Methods

### Registration

To describe patient characteristics and the process of care for patients with shoulder syndromes, data were used from the Netherlands Information Network of General Practice (LINH) [2] and the National Information Service for Allied Health Care (LIPZ) [1]. At the start of LINH, a random sample was drawn from the human resources register of GPs. Participating GPs record data on all patient contacts, including diagnoses, referrals and prescriptions. LIPZ is a registration network of physiotherapy practices that collects healthcare-related information on patient characteristics, mode of access, health problems and treatment plans on an ongoing basis. At the start of LIPZ, a random sample was drawn from the human resources register of physiotherapists.

According to Dutch legislation entitled ‘Regulations on medical research involving human subjects’, ethical approval is required for medical research in which persons are subjected to treatment or are required to behave in a certain manner. As this was not the case for the present study, ethical approval was not necessary. Nevertheless, the Dutch Data Protection Authority was notified of the research. In addition, pursuant to the Personal Data Protection Act, data were collected anonymously, patients were informed about the research by posters and leaflets in practice waiting rooms and patients had the opportunity to refuse participation. The research was carried out in accordance with the Helsinki Declaration.

### Participants

From the LINH database, all patients with shoulder syndromes, ICPC L92 (International Classification of Primary Care [18]) who visited the GP in 2008 (n = 2428) were selected. Eighty-five LINH practices were included, providing a representative sample of Dutch general practices with regard to practice type (solo, dual, group or health centre), degree of urbanisation and region. Patients with ICPC L92 were also selected from the LIPZ database. Because this database is smaller, all patients who visited the physiotherapist between 2006 and 2010 (n = 1182) were selected. Forty-nine LIPZ practices were included and 116 physiotherapists delivered data, providing a representative sample of Dutch physiotherapy practices with regard to practice type (solo, dual, group), degree of urbanisation and region. This is also true for the selection of physiotherapists with regard to age and year of graduation but there are more male therapists (p = 0.01) that register for LIPZ and the number of direct patient-related working hours is higher (p = 0.05).

### Data collection

In LINH, for every patient, a care episode was defined as the time between the first and last visit for L92 in 2008. Care episodes were constructed on the basis of EPICON, which is an algorithm that groups ICPC-coded contact records from electronic medical records in general practice into care episodes. This algorithm calculates care episodes for each year separately (Biermans et al., 2008). Prescriptions were registered in accordance with the Anatomical Therapeutic Chemical (ATC) classification system. Because not all prescriptions were linked to a diagnosis, a list was made of the most common prescriptions based on a group of patients with a known diagnosis of L92. For each of these prescriptions, the number of times they were prescribed during a shoulder-syndrome care episode was determined. Interventions were registered using CTG codes, which are standardised codes set by the Dutch healthcare authority for health care claims to health insurers. For the most common codes with a diagnosis of L92, the number of times they occurred during a care episode of shoulder syndrome was determined. Referrals were also registered and for the most common referrals with a diagnosis of L92, the number of times they occurred during a care episode of shoulder syndromes was established. Based on the information in the referral module, the total group of patients was split in two: patients referred for physiotherapy during the care episode versus patients who were not referred for physiotherapy.

In LIPZ, a series of consecutive treatment sessions for shoulder syndromes was considered to be a care episode. For each care episode, the gender and age of the patient was registered. Also recorded were the duration of the complaint at intake, whether it was a recurrent complaint (when the complaint appeared after a symptom-free period of at least four weeks and at most two years) and the treatment goal(s). At the end of the treatment, therapists registered a maximum of three interventions that were applied in at least 50% of the treatment sessions. Besides these features of the treatment plan, the outcome was also registered (indication of the extent to which the treatment goals were met, according to the physiotherapist). Based on the mode of access, the total group of patients was split in two: patients referred by their GP versus patients who accessed physiotherapy directly.

### Data analysis

Descriptive statistics were calculated for all variables using Stata 11. Chi-square tests (a = 0.05) were used to test differences in categorical data between patients with shoulder syndromes with and without a referral; two-sample t-tests were used for continuous data.

## Results

Incidence of shoulder syndromes in general practice in 2008 was 8.5 patients per 1000 patients, or 38% of all shoulder complaints. Prevalence was 14.2/1000 patients/year, or 42% of all shoulder complaints. GPs treated 82% (n = 1983) of patients themselves and referred 18% (n = 445) to one or more other clinicians, mainly to a physiotherapist (13%, n = 306) or a medical specialist (7%, n = 165) (total is more than 18% because there may have been more than one referral per patient). In two-thirds of the referred patients (n = 199), the referral for physiotherapy was given during the first GP consultation without further treatment by the GP, seven percent (n = 20) were referred within two weeks and a further seven percent (n = 22) within one month. In general practice, there was no difference in terms of age or gender between patients who were referred for physiotherapy and those who were not (Table 1); 42% (n = 1016) of the patients with shoulder syndromes were male and the mean age was 55 years (SD 15).

**Table 1:** Patient characteristics in general practice and physiotherapy practice

	***General practice *( *GP *)**		***Physiotherapy practice *( *PT *)**	
	***Only GP***	***GP *=>*PT***	***GP *=>*PT***	***Only PT***
	**(n = 2134)**	**(n = 294)**	**(*n *= *895*)**	**( *n *= *148 *)**
Mean age (years ± SD) †	55 (15)	55 (14)	57 (16)	53 (16)
Gender (% male) †	42	44	41	54

† *Significant difference between* ‘*only PT patients*’ *in PT and* ‘*GP* =>*PT patients*’ *in PT*.

Table 2 shows that treatment in general practice was different for patients who were referred for physiotherapy. They consulted their GP more often but received less medication; in particular, fewer patients were prescribed NSAIDs. Of the patients referred for physiotherapy, 37% (n = 109) consulted their GP again after the visit during which the referral was made.

**Table 2:** Treatment characteristics in general practice

	***General practice *( *GP *)**	
	***Only GP *( *n *= *2134 *)**	***GP *=>*PT *( *n *= *294 *)**
Prescriptions (%)†	69	50
Paracetamol†	4	8
NSAID†	50	38
Corticosteroids	24	19
Local anaesthetic	11	9
Opioids	7	7
Interventions		
Consultation (mean number ± SD)†	1.5 (1.4)	2.0 (1.6)
Duration of care episode (in days)†	46	60
Cyriax injection (%)†	29	21

† *Significant difference between* ‘*only GP patients*’ *and* ‘*GP* =>*PT patients*’.

In physiotherapy practice, 2.6% (n = 1182) of all patients presented with shoulder syndromes, accounting for 27% (n = 1182) of all shoulder complaints. Of these, 76% (n = 895) were referred by a GP, 12% (n = 139) by a medical specialist and 12% (n = 148) accessed the service directly. Self-referrals differed from referred patients; they were younger and more often male (Table 1). Furthermore, they more often had recurrent problems and these were more frequently related to sports and leisure activities and less often to work (Table 3). The treatment also differed; in self-referrals, treatment goals were more often aimed at muscle function.

**Table 3:** Complaint and treatment characteristics in physiotherapy practice

	***Physiotherapy practice *( *PT *)**	
	***GP *=>*PT *( *n *= *895 *)**	***Only PT *( *n *= *148 *)**
Recurrent complaint (% yes) †	22	31
Duration of complaints (%)		
<1 month	30	33
Interventions1-3 months	34	36
>3 months	36	31
Previous physiotherapy (% yes)	44	47
Pain severity (NRS 0–10) (mean ± SD) (n = 210/57) *	7 (1.7)	7 (1.1)
Onset (%) (n = 238/62)*		
Gradual	76	71
Sudden	24	29
Cause† (%) (n = 158/44)*		
Sport	13	41
Work	33	27
Leisure activities	13	18
Other	41	14
Treatment sessions (mean ± SD)	15 (18)	15 (19)
Duration of treatment (mean ± SD, in weeks)	13 (15)	12 (15)
Treatment goals (%)†		
Mobility	47	33
Muscle function	11	20
Pain	11	13
Other	31	34
Interventions (% used in ≥50% of the treatment sessions)		
Mobilisation	45	39
Massage	39	45
Physical agent modalities	12	13
Exercise therapy – functions†	70	55
Exercise therapy – skills	24	32
Information & advice	33	33
Treatment goals fully reached (%)	63	69

† *Significant difference between* ‘*only PT patients*’ *and* ‘*GP* =>*PT patients*’.

* *Registration since 2009*.

There were no differences between referred patients and self-referrals in terms of the duration of the complaint at the start of treatment, previous physiotherapy, severity of the complaint or the onset. Of patients with shoulder syndromes, 35% (n = 365) waited more than three months before visiting a physiotherapist, 45% (n = 469) had already had physiotherapy previously, severity of the complaint (between 0 and 10) was rated 7 and in 75% (n = 224) of the patients the symptoms had developed gradually. Common combinations of interventions were exercises aimed at functions and mobilisation or massage. At the end of treatment, the results did not differ between referred patients and self-referrals: in 64% (n = 668) of all patients with shoulder syndromes the treatment goals were fully reached.

## Discussion

The present study sought to determine the characteristics of the patient population and the types of treatment for patients with shoulder syndromes in both general practice and in physiotherapy practice and secondly, whether there are differences between patients who are treated by their GP, those who are referred for physiotherapy and those who access physiotherapy directly. The results show that there are differences between these populations both in terms of the characteristics of the patient and the treatment they receive.

Eight out of ten patients with shoulder syndromes that visit a GP are treated solely by the GP and not referred to another clinician. In these patients, treatment was aimed at reducing pain and inflammation. The number of patients referred for physiotherapy in the present study was comparable to that of Kuijpers et al. [19]. Most patients who received a referral for physiotherapy, were referred early on and were prescribed less medication. The guideline for shoulder complaints from the Dutch College of General Practitioners recommend a stepwise approach in which the patient is referred for physiotherapy when there is no improvement with rest and advice (‘wait and see’) and pain medication for one or two weeks (preferably paracetamol) [9]. When pain is the main problem, extended treatment with analgesia is indicated. Physiotherapy is mainly indicated in the presence of a limited range of motion or other functional limitations. Although the duration of the complaints does not appear on GP records, the high number of first visit referrals indicates a discrepancy between the guidelines and practice regarding the time frame for referral to physiotherapy. Further experimental research into the long-term effectiveness of early versus later referral is required to determine the preferred procedure. Duration of the complaints, level of pain, presence of functional limitations and concomitant cervical or cervicothoracic dysfunction will need to be taken into account.

In previous research, it was demonstrated that patients with shoulder complaints make as much use of direct access as the general patient population when attending the physiotherapist [17]. However, the results of this study show that patients with *shoulder syndromes* make less use of direct access; only 13% came through direct access compared with 22-44% of the entire patient population attending the physiotherapist from 2006 to 2010. It is known that self-referral decreases with age. The average age of patients with shoulder syndromes was 56 and therefore, the number of self-referrals can be expected to be lower in comparison with the general patient population in physiotherapy practice. Pain severity might also explain the limited number of self-referrals among patients with shoulder syndromes. Pain is common in shoulder syndromes and the average score on the numeric rating scale for pain severity was seven for both referred patients and self-referrals. Kennedy et al. found a comparable level of pain severity in patients with soft tissue disorders [8]. Given the type of treatment offered in general practice, patients with severe pain might turn to a GP first. The difference might also be related to the onset of pain. Self-referrals more often involve complaints of a short duration [17]. In three quarters of the patients with shoulder syndromes, the symptoms developed gradually; a much higher proportion than seen in the general patient population (60%) [1]. Van der Windt et al. [20] showed that a relatively large proportion of patients with shoulder syndromes considered strain or overuse in usual activities to be the precipitating cause of their problems. This study also shows that many patients wait a long time before they visit a physiotherapist. Kennedy et al. also found that almost half of patients with soft tissue disorders of the shoulder wait more than three months before contacting a physiotherapist [8]. It seems worthwhile to bring this information to the attention of patients since both a gradual onset and long-lasting complaints might contribute to an unfavourable prognosis [21]. However, earlier physiotherapy intervention for more patients is more expensive. It is the responsibility of the profession to act on this. The new guideline on shoulder syndromes advises physiotherapists to use the classification of pain and functional limitations, as practised by GPs. Given the limited value of clinical shoulder tests, even when combined [22], this could be a helpful approach. Perhaps with early detection, a once-off consultation during which advice is given will be sufficient. Regarding the use of such a wait-and-see policy by physiotherapists, the profession will need to determine the conditions under which this is possible as well as its impact on prognosis and cost-effectiveness.

With regard to the physiotherapy treatment itself, the results of the present study show that in patients with shoulder syndromes, exercises aimed at functions, mobilisation and massage are the main types of intervention, which is partly in line with what is known about the treatment of shoulder injuries. Literature reviews by Green et al. [12] and Kromer et al. [15] on physiotherapy interventions for shoulder pain did not mention massage, whereas other research on the effectiveness of massage for shoulder pain provided moderate evidence for analgesic effects.

Physiotherapy treatment results in a positive outcome in 64% of patients with shoulder syndromes, regardless of the mode of access. In the general patient population in physiotherapy practice, 68% fully reach the treatment goals [1]. Of the patients referred for physiotherapy, 37% go back to their GP. This is in line with previous studies indicating an unfavourable outcome in many patients resulting in high costs [3,23]. On the other hand, Kuijpers et al. found that the total costs in the six months after first consultation for shoulder pain in primary care were not alarmingly high. In that study, the cost of physiotherapy accounted for only 14% of the total costs, as few patients were referred for therapy. However, the authors concluded that higher health care costs and productivity losses may be expected when follow-up times are longer due to a poor prognosis [24].

Registration networks cover a large number of patients, providing a rich source of data. However, there are some limitations to this method of data collection. In LIPZ, information is collected on all diagnoses. This means detailed information specific to shoulder syndromes is not available; e.g. the existence of neck or back problems or repetitive or provocative movements in work or sport. Furthermore, diagnoses are based on referral letters, which can be ambiguous or imprecise. For example, terms such as ‘shoulder complaints’ are used, without giving further information. The procedure for diagnosing specific shoulder disorders is further complicated by a lack of consensus on the diagnostic criteria. Where diagnosis is difficult, complaints may be described as general shoulder complaints in the first instance, perhaps more so by less experienced clinicians. In this study, these general shoulder complaints are not included as shoulder syndromes in order to prevent heterogeneity as much as possible. Therefore, the results are based on a more homogeneous group of patients, but this may have led to an underestimation of the number of people attending the physiotherapist with shoulder syndromes. To measure the outcome of physiotherapy treatment, an indication of the extent to which the treatment goals were met is registered in LIPZ by the physiotherapist. This is a subjective outcome measure. In 2010, an indication of symptom severity at the beginning of the care episode and at the end was introduced. When patients do not come back, this information, which has to be obtained from the patient, remains unknown. As a result, this outcome measure is only known for a subgroup of patients, which is insufficient for a reliable investigation. Therefore, physiotherapists give an indication of the result, so that an outcome measure is known for every patient. In the present study, referred patients and self-referrals achieved the treatment goals to the same extent. Since the outcome is measured in the same subjective manner, it is not expected that the results would be different. Nevertheless, ideally, patient-reported outcome measures should also be studied.

In LINH, a diagnosis was not registered for every consultation. Prescriptions, referrals and interventions are calculated for the total care episode of shoulder syndromes and might therefore have been overestimated. However, this calculation only concerned a selection of frequently used prescriptions, referrals and interventions which prevents the inclusion of those actually relating to a diagnosis other than shoulder syndromes.

Finally, data is based on two different patient populations. The physiotherapy database is much smaller and, therefore, a longer time period was selected. However, there were no policy changes in the area or indications that the group of patients consulting their GP changed over the period of the study. Nevertheless, it would be interesting to investigate the care process in a multidisciplinary network incorporating the activities of various health care professionals.

## Conclusions

In summary, there are differences in general practice between patients who are referred for physiotherapy and those who are not. Patients who are referred are prescribed less medication and are often referred at the first consultation with their GP. This goes against current guideline for GPs and could result in unnecessary or higher costs. On the other hand, possibly due to the gradual onset of complaints and a wait-and-see policy, for many patients, it takes quite a while before they see a physiotherapist, even though it is suggested that a long duration of complaints could be a predictor for poorer outcomes. When a restricted range of motion is the main problem, it is arguable that patients receive less medication but a quick referral to a physiotherapist. Future research into the long term cost-effectiveness of an early referral could demonstrate whether this leads to better outcomes and should therefore be the preferred treatment.

As clinicians, we should also develop a way of educating patients about shoulder syndromes to prevent them waiting too long before they seek help. However, this can only be cost-effective when the profession sets clear guidelines on indications for physiotherapy, especially since there is debate on the value of clinical diagnostic tests. The classification of pain and functional limitations and adoption of the wait and see policy as used by GPs could be an example or starting point. Perhaps with early detection, a once-off consultation in which advice is given will be sufficient, especially when pain is severe. The consequences of such initiatives for the prognosis of the individual patient as well as cost-effectiveness should be investigated first.

## Abbreviations

GP: General Practitioner; LINH: Netherlands Information Network of General Practice; LIPZ: National Information Service for Allied Health Care; ICPC: International Classification of Primary Care; EPICON: An application to group ICPC-coded diagnoses from electronic medical records in general practice into episodes of care; ATC: Anatomical Therapeutic Chemical; CTG: Codes for claiming health care services to health insurers; PT: Physiotherapy Practice; NSAID: Non Steroidal Anti Inflammatory Drugs; NRS: Numeric Rating Scale; SD: Standard Deviation

## Competing interests

The authors declare that they have no competing interests.

## Authors’ contributions

MK contributed to conception and design, acquisition of data, analysis and interpretation of data and was involved in drafting the manuscript. IS contributed to conception and design, acquisition of data, analysis and interpretation of data and was involved in revising the manuscript critically. CD contributed to acquisition of data, analysis and interpretation of data and was involved in revising the manuscript critically. DB contributed to analysis and interpretation of data and was involved in revising it critically. CV contributed to conception and design, analysis and interpretation of data and was involved in revising the manuscript critically. All authors read and approved the final manuscript.

## Pre-publication history

The pre-publication history for this paper can be accessed here:

http://www.biomedcentral.com/1471-2474/14/128/prepub

## References

[B1] Kooijman MK Swinkels ICS Leemrijse CJ de Bakker DH Veenhof C National Information Service of Allied Health Care 2011:

[B2] Verheij RA van Dijk CE Stirbu-Wagner I Dorsman SA Visscher S Abrahamse H Netherlands Information Netwerk of General Practices 2011:

[B3] Windt DAWM Koes BW Boeke AJP Devillé W Jong BA Bouter LM Shoulder disorders in general practice: prognostic indicators of outcome Br J Gen Pract 1996 46 410 519 523 8917870 PMC1239746

[B4] Croft P Pope D Silman A The clinical course of shoulder pain: prospective cohort study in primary care Br Med J 1996 313 601 602 10.1136/bmj.313.7057.601 8806252 PMC2352021

[B5] Winters JC Sobel JS Groenier KH Arendzen JH Meyboom-de Jong B: The long-term course of shoulder complaints: a prospective study in general practice Rheumatology 1999 38 160 163 10.1093/rheumatology/38.2.160 10342630

[B6] Bot SDM Van der Waal JM Terwee CB Van Der Windt DAWM Scholten RJPM Bouter LM Predictors of outcome in neck and shoulder symptoms Spine 2005 30 16 E459 E470 10.1097/01.brs.0000174279.44855.02 16103840

[B7] Virta L Joranger P Brox JI Eriksson R Costs of shoulder pain and resource use in primary health care: a cost-of-illness study in Sweden BMC Musculoskelet Disord 2012 13 1 17 10.1186/1471-2474-13-17 22325050 PMC3299609

[B8] Kennedy CA Manno M Hogg-Johnsson S Haines T Hurley L McKenzie D Prognosis of soft tissue disorders of the shoulder: predicting both change in disability and level of disability after treatment Phys Ther 2006 86 7 1013 1032 16813480

[B9] Winters JC Van Der Windt DAWM Spinnewijn WEM de Jongh AD van der Heijden GJMG Buis PAJ Dutch College of General Practitioners' standard shoulder complaints Huisarts en Wetenschap 2008 51 11 555 565 10.1007/BF03086936

[B10] Hung C-J Jan M-H Lin Y-F Wang T-Q Lin J-J Scapular kinematics and impairment features for classifying patients with subacromial impingement syndrome Man Ther 2010 15 6 547 551 10.1016/j.math.2010.06.003 20615748

[B11] Dorresteijn O Stevens M Winters JC van der Meer K Diercks RL Conservative or surgical treatment for subacromial impingement syndrome? A systematic review J Shoulder Elbow Surg 2009 18 4 652 660 10.1016/j.jse.2009.01.010 19286397

[B12] Green S Buchbinder R 2003 Hetrick SE: Physiotherapy interventions for shoulder pain. Cochrane Database of Systematic Reviews (2)

[B13] Hegedus EJ Goode A Campbell S Morin A Tamaddoni M Moorman CT Physical examination tests of the shoulder: a systematic review with meta-analysis of individual tests Br J Sport Med 2008 42 2 80 92 10.1136/bjsm.2007.038406 17720798

[B14] Kelly SM Wrightson PA Meads CA Clinical outcomes of exercise in the management of subacromial impingement syndrome: a systematic review Clin Rehabil 2010 24 2 99 109 10.1177/0269215509342336 20103573

[B15] Kromer TO Tautenhahn UG de Bie RA Staal JB Bastiaenen CHG Effects of physiotherapy in patients with shoulder impingement syndrome: a systematic review of the literature J Rehabil Med 2009 41 11 870 880 10.2340/16501977-0453 19841837

[B16] Jansen MJ Brooijmans F Geraets JJXR Lenssen T Ottenheijm R Penning L Evidence based statement subcromial complaints Nederlands Tijdschrift voor Fysiotherapie 2011 121 1

[B17] Leemrijse CJ Swinkels ICS Veenhof C Direct access to physical therapy in the Netherlands: results from the first year in community based physical therapy Phys Ther 2008 88 8 936 946 10.2522/ptj.20070308 18566108

[B18] Lamberts H Wood M International Classification of Primary Care 1987 Oxford: Oxford University Press

[B19] Kuijpers T Van Der Windt DAWM Boeke AJP Twisk JWR Vergouwe Y Bouter LM Clinical prediction rules for the prognosis of shoulder pain in general practice Pain 2006 120 3 276 285 10.1016/j.pain.2005.11.004 16426760

[B20] Windt DAWM Koes BW, de Jong BA, Bouter LM: Shoulder disorders in general practice: incidence, patient characteristics, and management Ann Rheum Dis 1995 54 12 959 964 10.1136/ard.54.12.959 8546527 PMC1010060

[B21] Kuijpers T Van Der Windt DA Van Der Heijden GJ Bouter L Systematic review of prognostic cohort studies on shoulder disorders Pain 2004 109 3 420 431 10.1016/j.pain.2004.02.017 15157703

[B22] Hegedus EJ Goode AP Cook CE Michener L Myer CA Myer DM Which physical examination tests provide clinicians with the most value when examining the shoulder? Update of a systematic review with meta-analysis of individual tests Br J Sport Med 2012 46 14 964 978 10.1136/bjsports-2012-091066 22773322

[B23] Nygren A Von Koch M Neck and shoulder pain: an increasing problem Scand J Rehabil Med 1995 32 107 112 7784832

[B24] Kuijpers T van Tulder MW Van Der Heijden GJ Bouter LM Van Der Windt DAWM Costs of shoulder pain in primary care consulters: a prospective cohort study in the Netherlands BMC Musculoskelet Disord 2006 7 83 10.1186/1471-2474-7-83 17078883 PMC1635047

